# The Correlation between Hormonal Disturbance in PCOS Women and Serum Level of Kisspeptin

**DOI:** 10.1155/2020/6237141

**Published:** 2020-01-07

**Authors:** Razaw O. Ibrahim, Shirwan H. Omer, Chro N. Fattah

**Affiliations:** ^1^Department of Physiology, College of Medicine, University of Kirkuk, Kirkuk, Iraq; ^2^Department of Physiology, College of Medicine, University of Sulaimani, Sulaymaniyah, Iraq; ^3^Department of Obstetrics and Gynecology, College of Medicine, University of Sulaimani, Sulaymaniyah, Iraq

## Abstract

**Background:**

Kisspeptin is a neuropeptide that upregulates gonadotropin-releasing hormone (GnRH) secretion. It is an essential element for the luteinizing hormone (LH) surge and ovulation. Women with polycystic ovary syndrome (PCOS) expose alteration in both GnRH and LH secretion levels.

**Objective:**

This paper aims to evaluate serum kisspeptin levels in healthy and polycystic ovarian syndrome women. Furthermore, it investigates the effect of obesity and age on circulating kisspeptin levels in both normal and PCOS women. Moreover, it points out the correlation between kisspeptin and other hormonal parameters. *Methods and Patients*. One hundred women (60 are with PCOS and 40 are normal) were enrolled in the study. Five milliliter samples of blood from all the patients and control women were obtained twice during the menstrual cycle. All the study samples were classified depending on the age factor for several subgroups.

**Results:**

Kisspeptin levels were higher in PCOS patients than those in the normal group. Kisspeptin correlated with serum free testosterone level (*r*=0.26). In healthy women, preovulatory kisspeptin levels were higher than follicular kisspeptin levels (*P* < 0.05), while this difference was insignificant in PCOS patients. The variation in serum kisspeptin levels between overweight/obese and normal-weight women was insignificant. In normal women, serum kisspeptin levels were higher in women >35 years than those <24 years at (*P*=0.03).

**Conclusion:**

The serum kisspeptin level is higher in PCOS women. Its levels fluctuate during the menstrual cycle, but these fluctuations are disturbed in PCOS women. The effect of BMI on serum kisspeptin levels is insignificant, and kisspeptin serum levels increase with age.

## 1. Introduction

Recently, it has been documented that hypothalamic kisspeptin acts as a stimulator of GnRH and mediates sex hormone feedbacks. This neuropeptide has great importance at the onset of puberty and maintenance of normal reproductive function [1]. Kisspeptin is composed of several amidated peptides that are formed due to the differential proteolytic process derived from (145) amino acids and encoded by the kisspeptin gene (KISS1 gene) [2]. Other peptide fragments of the kisspeptin precursor, such as kisspeptin-14, kisspeptin-13, and kisspeptin-10, have the same COOH-terminal region of kisspeptin. It is worth mentioning that all types of kisspeptin act collectively via the G protein-coupled receptor GPR54 [3]. The kiss1 gene located in chromosome 1q32 consists of three exons, in which only part of the second and third exons is finally translated into the precursor 145 amino acid peptide, which is cleaved into three mentioned forms of kisspeptin [4].

It is well-known that the kisspeptin location varies according to the species type. In humans, the receptor ligand (*KISS1* mRNA) has been found in the placenta, testis, pancreas, liver, and small intestine [5]. The hypothalamus and KISS1 neurons are present predominantly in the infundibular nucleus (which is the homolog of the arcuate (ARC) in rodents and some other animals) and in separated foci in the medial preoptic area [5, 6]. In rats and mice, there are two major areas in the hypothalamus that contain kisspeptin neurons, which are anteroventral periventricular (AVPV) nucleus (the rostral one) and ARC (the caudal one), which contain more kisspeptin neurons [7, 8].

In normal mice, estradiol's negative feedback increases glutamatergic transmission to ARC kisspeptin neurons and decrease it to AVPV neurons, while estradiol positive feedback had the antipode effect [9]. Estradiol elevation exits the AVPV kisspeptin neurons by increasing the number of depolarization-induced bursts and rebound bursts [10]. Knocked alpha estradiol receptor in the ARC kisspeptin neurons tends to associate with cycle disruption, where, knocked ER*α* in AVPV kisspeptin neurons tends to associate with the normal cycle, but blunted the LH surge [8]. In humans, both negative and positive kisspeptin feedbacks occur in the infundibular nucleus. Yet, KISS1 expression in the infundibular nucleus has been shown to increase after menopause [8, 11]. This evidence strongly supports the kisspeptin role in reproductive physiology.

The common cause of ovulatory subfertility is polycystic ovarian syndrome. Women with this syndrome also display menstrual irregularity, hair growth, acne, and overweight. However, it is frequently seen in normal-weight women. Alteration in GnRH secretion is a feature of PCOS. GnRH expresses slow and fast pulse generation for stimulation of FSH and LH, respectively. In PCOS women, LH commonly increases. FSH is typically in a lower range. This may be related to decrease the sensitivity of the GnRH pulse generator to steroid feedbacks and enhance LH secretion [12]. The elevated level of GnRH and LH in PCOS women may be related to the cumulative effect of altered GnRH stimulatory and inhibitory neurotransmitters in the hypothalamic-pituitary center [13]. The detailed mechanism for how the kisspeptin is involved in the pathogenesis of PCOS is still unknown. However, recently it has been proven that metabolic disorders in PCOS women may contribute to the alteration of the kisspeptin level [14–16].

The role of kisspeptin in conveying metabolic signals to brain centers had been under the spotlight. Lacking kisspeptin signaling in female mice causes an increase in body weight, adiposity, and glucose intolerance [17]. In women, a positive correlation between BMI and KISS1 expression was found in omental adipose tissue [18].

Finally, this study aims to investigate the effect of age and obesity on circulating kisspeptin levels in healthy and PCOS infertile women. Additionally, it examines the correlation between serum kisspeptin and other biochemical markers.

## 2. Materials and Methods

### 2.1. Subjects

The study samples were collected from the family planning center (Infertility Clinic), during the period from April 2018 to March 2019. The study selected 100 women (aged 20 to 40) and classified them into two groups. First, 60 patients who were infertile and unable to conceive after a year and six months of regular unprotected intercourse for those aged (<35) and (>35), respectively. Second, 40 patients were fertile with the regular menstrual cycle. The 60 infertile patients were diagnosed as polycystic ovarian syndrome. The diagnosis was based on the Rotterdam consensus meeting on PCOS in 2003 [19]. Menstrual disturbance involved amenorrhea marked by an absence of menstrual cycle for more than 6 months and oligomenorrhea marked by a delay in the menses of >35 days for 6 months. The transvaginal ultrasonic screening was performed twice for all infertile women. Ovaries are described as polycystic ovaries with 12 or more follicles (2–9) mm in one or both ovaries. The second transvaginal ultrasound was performed on the 12^th^, 13^th^, and 14^th^ day of the cycle for detecting mature follicle (more than 16 mm) and ovulation.

Hysterosalpingography, seminal fluid analysis, laparoscopy, and postcoital tests were performed for all infertile women. The patients with abnormal ovulatory assessment were excluded from the study. Exclusion criteria were as follows:

1. None of the patients took any medication for at least (3) months before participating in the study.
2. Abnormal thyroid function test, elevated prolactin level, Cushing syndrome, and inability to follow-up. Before starting the study, ethical permissions were obtained. Five milliliters of blood were taken from the participants (who visited the clinic for consultation) after their agreement to participate in the study.

The PCOS patients were subdivided into four subgroups (according to the clinical and biochemical markers) as follows:

  Group A: ovulatory dysfunction + hirsutism or hyperandrogenism + PCO feature
  Group B: ovulatory dysfunction + hirsutism or hyperandrogenism
  Group C: ovulatory dysfunction + PCOS (no hirsutism and normal androgen)
  Group D: hirsutism or hyperandrogenism + PCOS with the normal menstrual cycle

The fertile and infertile PCOS women were subdivided into four subgroups according to their ages:

1. 20–24 years old
2. 25–29 years old
3. 30–33 years old
4. 35–40 years old

### 2.2. Test Protocol

Five milliliters of blood was obtained twice from all patients and the study groups by vein puncture. The first samples were taken on day 2 of the cycle. The second samples (in fertile women) were obtained at a preovulatory phase in the days (12^th^, 13^th^, and 14^th^) of the period depending on detecting premature follicles. However, in PCOS patients, the second samples were obtained on day 13 of the same cycle. After that, all the samples were incubated at room temperature for two hours for completing the clotting process. Next, the serum was separated by centrifugation for 20 minutes at 3000 rpm. Then, it was transferred to plain tubes and stored at 20°C until the assay process. Finally, all the parameters were measured by enzyme-linked immunosorbent assay (ELISA).

### 2.3. Statistical Analysis

In this study, the values presented as mean ± SD and the Kolmogorov–Smirnov test was used to test the normality of distribution. Student's *t*-test was used to compare the two group's means. A one-way analysis of variance (ANOVA) was performed to estimate the differences between the groups. Then, Tukey's posthoc test was used to evaluate the relationship between the two groups. Lastly, the calculation of correlation and Pearson correlation were performed for assaying the correlation between kisspeptin and other biochemical markers in both diseased and healthy women, respectively.

## 3. Results

As listed in Table 1, there were significant differences in kisspeptin, estradiol, free testosterone, and FSH and LH levels between PCOS (infertile) and control women (*P* ≤ 0.05).

**Figure 1:**
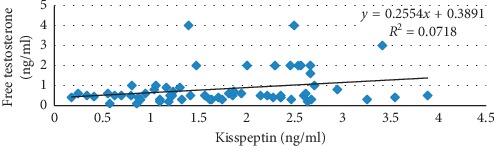
The correlation between free testosterone and kisspeptin (*r*=0.26) in infertile PCOS women.

As listed in Table 3, there was a positive correlation between serum kisspeptin levels and serum free testosterone (*r*=0.26; *P*=0.04). Furthermore, the results demonstrated no correlation between kisspeptin and other hormonal parameters. These findings are shown in Table 3 and Figure 1.

As outlined in Table 4, in PCOS infertile women, the difference between serum kisspeptin levels at two different phases of the same was not significant. On the other hand, in normal women, the kisspeptin level in the preovulatory phase was significantly higher than what it was in the follicular phase.

There were no significant differences in serum kisspeptin levels between overweight/obese group and nonobese PCOS patients (see Tables 5–7). Furthermore, insignificant difference between overweight/obese and nonobese healthy women was obtained. On the other hand, their levels in nonobese PCOS patients were higher than those in nonobese fertile women.

As outlined in Table 8, the infertile PCOS women were subdivided into four subgroups according to their ages. There were no significant variations in kisspeptin levels in serum among the age subgroups (*F*=0.128; *P*=0.924). Furthermore, there were no significant changes in kisspeptin between age subgroups.

Unlike infertile PCOS women, the kisspeptin levels showed significant variations among age subgroups in fertile women (*F*=3.2; *P*=0.03). Furthermore, kisspeptin levels were significantly higher in the first group as compared to group 4 (*P*=0.0166) (see Table 9).

## 4. Discussion

In this study, the follicular phase level of the serum kisspeptin was significantly higher in infertile PCOS women as compared with the normal women (Table 1). In agreement with our result, increased serum kisspeptin levels in PCOS patients were observed in many studies, such as [14–21]. Other studies did not obtain this variation [22–25]. Panidis et al. [22, 25] used different PCOS diagnostic criteria. There was a significant variation in age and BMI in Albalawi et al. study. Panidis et al. study was conducted on a small sample size and explained why the insignificant variation was obtained. Recently, the discovery of kisspeptin and its receptor paved a way for investigations about its role in the pathogenesis of PCOS. It is well known that the kisspeptin causes upregulation of GnRH. Also, it exists in the ovary and is involved in ovulation and sex hormone regulation. Moreover, alteration in kisspeptin secretion might result in a considerable commotion of the gonadotropic axis similar to that present in PCOS patients. As the PCOS is a heterogeneous syndrome, not all patients share the same endocrine and hormonal alterations. We hypothesized that the serum kisspeptin level may be higher in phenotypes with ovulation dysfunction and hyperandrogenism. As mentioned earlier, the infertile PCOS patients were divided into four subgroups according to the Rotterdam criteria. In this study, most PCOS patients have ovulatory dysfunction and hyperandrogenism (Table 2). Recently, a variety of experimental animal studies have been conducted to estimate KISS1, KISS *r*, and KISS-positive cells in different PCOS-induced phenotypes. Aliabadi et al. found that [26, 27] kisspeptin-positive cells and kiss mRNA expression in letrozol-injected female rats were higher than normal rats. Additionally, they demonstrated that the enhanced neural cells in PCOS rats might contribute to the hypersecretion of LH. Similarly, Kondo et al. [28] who studied ARC kisspeptin immune reactivity by using antiprogestin R4486 found that besides the increase in the LH level, there was also an increase in the number of kisspeptin-positive cells in the hypothalamus. On the contrary, Marcondes et al. [29, 30] did not agree with this. They suggested that low kisspeptin expression in testosterone-treated rats might contribute to anovulation and decrease in LH secretion. However, Osuka et al. [31] found that rats treated by dihydrotestosterone showed normal LH levels. These inconsistencies in the effect of androgens on the kisspeptin expression are partly because of different methods employed to evaluate this impact. Besides, the difference in the age of experimental animals may contribute to this controversy. In almost all previously mentioned studies that evaluated kisspeptin in different PCOS animal phenotypes, increased kisspeptin in the hypothalamus is not observed in all phenotypes. It is only observed in the phenotypes with high LH levels. This is in line with our hypothesis that kisspeptin levels may show variation among PCOS phenotypes.

As shown in Figure 1, kisspeptin level correlates significantly with only free testosterone level (*r*=0.26; *P*=0.04) (see also Table 3). Gorkem et al. [16] also found a positive correlation between kisspeptin and testosterone. Peripheral administration of kisspeptin in adult mice caused a dramatic increase in free testosterone level [6]. On the contrary, Iwata et al. [30] found that hyperandrogenism in adult rats caused a decrease in the number of KISS1-expressing cells in both AVPV and ARC nucleus. Additionally, they argued that the ARC nucleus (containing estrogen receptor) also has an androgen receptor which can be affected by excess androgen and so cause negative feedback to kisspeptin. Many pieces of evidence in humans and animals found that the hyperandrogenism leads to an increase of LH/GnRH secretion [32–35]. In Iwata et al. study [30], dihydrotestosterone was used, which may act through the estrogen negative pathway, not through the androgen pathway.

In healthy women, there were significant variations in serum kisspeptin levels in the preovulatory phase as compared with the follicular phase. Nevertheless, this difference was not quite significant in subfertile PCOS women (Table 4). In a normal monthly reproductive cycle, there is an initial rise in FSH level that causes gradual follicular maturation. Before the follicles grow, estrogen may suppress the kisspeptin level at this stage. This negative feedback changes to positive feedback when estrogen further increases with the LH surge. It is well known that kisspeptin is an essential element in producing LH surge and ovulation. Furthermore, its expression increases just before ovulation and LH surge [36–38]. Subcutaneous injection of kisspeptin-54 in women with hypothalamic amenorrhea caused a potential increase in LH and FSH levels. This response becomes clear in the preovulatory phase [39–41]. The effect of kisspeptin administration in women with or without steroid supplements was investigated in another study [42]. The authors found that more potent impact was observed in postmenopausal women with no estrogen supplements, suggesting the involvement of estrogen in negative kisspeptin suppression.

Similar to our findings, Latif and Rafique [43] also found that serum kisspeptin in healthy young women was significantly higher in the preovulatory phase. Regarding PCOS patients, the difference in serum kisspeptin levels at two different phases of the same cycle was not quite significant (*P*=0.07) (Table 4). The most common PCOS complication is anovulation; most of the PCOS women suffer from oligomenorrhea. Besides, there is a disturbance in GnRH pulse secretion followed by a disturbance in LH and FSH secretion. This study is better from others because of 2 blood collections from all patients. Recently, two studies [44, 45] evaluated the pairing between LH and kisspeptin in both PCOS and normal women. They found that in normal women, each kisspeptin pulse frequency is followed by the LH pulse frequency. Nevertheless, this rhythm was not observed in PCOS women, where kisspeptin and LH were increased independently. Additionally, however, they suggest that this irregularity is worse when the disease progresses. Finally, from the findings, we infer that the metabolic and endocrine disturbance that affected the follicular phase kisspeptin level could also affect its level in other phases of the cycle.

In the present study, we found that the difference in serum levels of kisspeptin in overweight/obese and normal-weight fertile women was insignificant (Table 6). This finding agrees with Rafique and Latif [46] who obtained the same result in normal and overweight Saudi women. Similar to previous findings, our results were also in line with Pita et al. [47] who did not find any correlation between BMI and kisspeptin. On the contrary, Kołoziejski et al. [48] found that the level of kisspeptin was significantly higher in normal-weight women as compared to obese women. They also demonstrated that there was a positive correlation between BMI and kisspeptin. One of the reasons for this decrement may be that the obese women in the Kołozeiejski et al. study [48] were with a BMI greater than 30. In this study, the BMI of both groups was less than 30 (Table 1). Human reproductive function was affected by extreme nutrition, under nutrition, and obesity. The kisspeptin conveys metabolic information into the brain centers that are responsible for reproductive regulation. Additionally, kisspeptin receptors are present in nonGnRH brain areas and peripheral tissue like adipose tissue. It has been experimentally proven that the expression of kiss mRNA and GnRH secretion can be reduced in fasting mice. Furthermore, a high-fat diet increases its level [49, 50]. Kisspeptin level may be affected by other metabolic and hormonal disorders that accompany obesity, for instance leptin level. The leptin level increases with increasing body fat. The relation between kisspeptin and leptin had been under focus where female mice lacking kisspeptin signaling displayed higher BMI and leptin levels. Additionally, a mutation in the leptin receptor also caused hypogonadism in humans [17, 51]. The leptin-responsive GABAergic neuron may regulate reproduction function by conveying signals of energy balance through kisspeptin neurons. GnRH neurons do not express leptin receptors, but they found that kisspeptin neurons express these receptors [52]. In the same perspective, Rafiq and Latif returned the association between obesity and kisspeptin to alteration in plasma triglyceride levels. They believe that hypertriglycemia induced lipotoxic inflammation in the hypothalamus. For estimating the relation between serum kisspeptin and obesity, a large sample size is needed. Furthermore, the study population should be divided according to BMI into underweight, normal weight, overweight, and obese women.

There was no statistical difference in kisspeptin levels between overweight and nonobese PCOS women (Table 5). This finding was enhanced by several previous studies, such as [15, 21, 23]. They found no relationship between BMI and kisspeptin in PCOS patients. However, Panidis et al. [22] demonstrated that kisspeptin in PCOS women was negatively correlated with BMI and insulin resistance. The number of PCOS women in the Panidis study was only 19. We believe that the correct estimation cannot be driven by this small number. In this study, the level of kisspeptin in nonobese PCOS patients was significantly higher than nonobese healthy women, suggesting that PCOS is the main etiological factor in raising serum kisspeptin (Table 7).

In the present study, both fertile and subfertile women were divided into four age groups. In the control women, the serum follicular phase kisspeptin level was significantly higher in subgroup 4 aged 35–40 as compared with subgroup 1 aged 20–24 (see Table 6).

No study compared serum kisspeptin among different age groups in healthy premenopausal women. To estimate the impact of age on the kisspeptin level and its neurons, several animal studies were conducted. After ovary removal in lab animals, the neurons that secrete KISS1, estrogen receptor mRNA in the arcuate nucleus hypertrophied, which causes an increment in the kisspeptin secretion [53–56].

In this study, the elevation of serum kisspeptin levels was observed in women below the age of 40. Several factors affect the reproductive age in women, for instance time of menarche, genetic factors, gravidity, and lactation. In spite of all healthy women who were with normal menstruation and normal FSH level, fertility and hormonal changes were observed in the premenopausal period. The level of estrogen may be normal or increased at this period [57]. We expect that women older than 40, may show a further serum kisspeptin rise, and many factors may lead to that. The most important factors are that the kisspeptinogeneic cells in the infundibular nucleus hypertrophied at this age. Also, dynorphin-secreting mRNA neurons are decreased [11]. The increase in kisspeptin levels may occur due to the lack of estrogen feedback [58]. Other studies proposed that this increase is related to an increase in ovarian sympathetic and adrenergic stimulation that occurs with aging and cause inovulation and kisspeptin elevation [59–61].

There were no statistical changes in serum kisspeptin levels in different age groups in PCOS patients (Table 7). These findings are in accordance with [15, 16, 24] which did not observe any correlation between age and kisspeptin in PCOS patients. The endocrine and hormonal alterations in PCOS patients may bestrew the normal changes in kisspeptin that occur with ovarian aging.

In summary, kisspeptin is higher in subfertile PCOS women. Kisspeptin involves in feedback mechanisms, and a disturbance in the reproductive axis could disturb the kisspeptin normal rhythm. Although kisspeptin did not correlated with the serum LH level, it correlated with the testosterone level, which is a common feature of PCOS. Kisspeptin serum levels could show variations among PCOS phenotypes, but for the accurate estimation, a larger study is recommended. Similar to gonadotropin hormones, kisspeptin serum level increases with advancing age.

## Figures and Tables

**Table 1:** The comparison of the biochemical markers, age, and BMI in infertile PCOS and control women.

Parameters	Infertile PCOS women (mean and SD)	Control (mean and SD)	*P* value
Number of the subjects	60	40	
Age (years)	30.09 ± 7.60	31.6 ± 7.16	NS
BMI (kg/m^2^)	26.05 ± 3.76	25.93 ± 3.7	NS
Kisspeptin (ng/ml)	1.79 ± 0.98	1.05 ± 0.86	<0.05
SHBG (nmol/L)	77.86 ± 55	80 ± 13.2	NS
Free testosterone (ng/ml)	0.66 ± 0.75	0.37 ± 0.22	<0.05
Estrogen (pg/ml)	47.76 ± 42	45.85 ± 15	<0.05
FSH (mIU/ml)	5.04 ± 1.33	6.25 ± 2	<0.05
LH (mIU/ml)	8.92 ± 5.47	4.57 ± 1.02	<0.05

The PCOS women were also subdivided into four phenotypes according to their clinical and biochemical parameters. The frequency of each phenotype is shown in Table 2.

**Table 2:** The frequency of PCOS patients' subgroups.

PCOS subgroups	Number of the patients	%
A	28	46.7
B	7	11.6
C	16	26.7
D	9	15

**Table 3:** The correlation between kisspeptin with other biochemical markers.

Parameters	Number	*P* value
BMI (kg/m^2^)	−0.13	0.30
FSH (mIU/ml)	−0.01	0.9
LH (mIU/Ml)	0.02	0.8
Free testosterone (ng/ml)	0.26	0.04
Estrogen (pg/ml)	0.145	0.26
SHBG (nmol/l)	0.078	0.58

**Table 4:** The comparison between serum kisspeptin levels at two different phases of the same cycle in both infertile PCOS and control women.

Infertile (PCOS) women		Fertile (control) women	
Menstrual cycle phase	Serum kisspeptin (ng/ml) (mean and SD)	Menstrual cycle phase	Serum kisspeptin (ng/ml) (mean and SD)
Follicular phase	1.79 ± 0.98	Follicular phase	1.05 ± 0.88
Preovulatory phase	2.1 ± 0.9	Preovulatory phase	1.56 ± 0.7
*P* value	>0.05	*P* value	<0.05

**Table 5:** The comparison in serum (kisspeptin, estrogen, and free testosterone) between overweight/obese and nonobese PCOS infertile women.

Parameters	PCOS women with BMI ≥ 25	PCOS women with BMI < 25	*P* value
Number of the subjects	37	23	
Kisspeptin (ng/ml)	1.59 ± 0.82	1.89 ± 0.9	NS
Estrogen (pg/ml)	41.38 ± 30.8	52.25 ± 34	NS
Free testosterone (ng/ml)	0.67 ± 0.54	0.81 ± 1.23	NS

**Table 6:** The comparison in serum (kisspeptin, estrogen, and free testosterone) between overweight/obese and nonobese fertile women.

Parameters	Fertile women with BMI ≥ 25	Fertile women with BMI > 25	*P* value
Number of the subjects	23	17	
Kisspeptin (ng/ml)	1.38 ± 1.2	0.84 ± 0.9	NS
Estrogen (pg/ml)	77.1 ± 23	45 ± 30.1	<0.05
Free testosterone (ng/ml)	0.25 ± 0.31	0.31 ± 0.28	NS

**Table 7:** The comparison in serum (kisspeptin, estrogen, and free testosterone) in normal-weight fertile and infertile PCOS women.

Parameters	PCOS women BMI < 25 kg/m^2^	Fertile women BMI < 25 kg/m^2^	*P* value
Number of the subjects	23	17	
Kisspeptin (ng/ml)	1.89 ± 0.9	0.84 ± 0.9	0.008
Estrogen (pg/ml)	52.25 ± 34	45 ± 30.1	NS
Free testosterone (ng/ml)	0.81 ± 1	0.31 ± 0.28	NS

**Table 8:** The serum kisspeptin level in different age groups in PCOS infertile women.

Age groups (years)	Number	Kisspeptin level (ng/ml) (mean and SD)	*P* value
Subgroup 1: 20–24	16	1.68 ± 0.71	1 vs 2 NS 1 vs 3 NS
Subgroup 2: 25–29	13	1.71 ± 0.78	2 vs 3 NS
Subgroup 3: 30–34	14	1.74 ± 0.80	3 vs 4 NS
Subgroup 4: 35–40	17	1.88 ± 1.19	1 vs 4 NS 2 vs 4 NS

**Table 9:** The serum kisspeptin levels in different age groups in fertile women.

Age groups (years)	Number	Kisspeptin level (ng/ml) (mean and SD)	*P* value
Subgroup 1: (20–24)	11	1.03 ± 0.81	1 vs 2 NS 1 vs 3 NS
Subgroup 2: (25–29)	10	1.2 ± 0.7	2 vs 3 NS
Subgroup 3: (30–34)	9	1.3 ± 0.3	3 vs 4 NS
Subgroup 4: (35–40)	11	2.01 ± 0.9	1 vs 4 *P*=0.0166

## Data Availability

All the data are presented in the manuscript.
